# Dental Students’ Knowledge and Attitudes towards Antibiotic Prescribing Guidelines in Riyadh, Saudi Arabia

**DOI:** 10.3390/pharmacy6020042

**Published:** 2018-05-07

**Authors:** Abdulrahman AboAlSamh, Abdulmalik Alhussain, Nawaf Alanazi, Rakan Alahmari, Naila Shaheen, Abdallah Adlan

**Affiliations:** 1 College of Dentistry, Riyadh Elm University (REU), Riyadh 13244, Saudi Arabia; Abdulmalik.ahussain@gmail.com; 2 College of Dentistry, Al Jouf University (JU), Sakakah 72388, Saudi Arabia; Alanazin5@gmail.com; 3 College of Dentistry, King Khalid University (KKU), Abha 62529, Saudi Arabia; Dr.rakan17@gmail.com; 4 King Abdullah International Medical Research Center (KAIMRC), Riyadh 14611, Saudi Arabia; ashrafna@ngha.med.sa (N.S.); Adlan@ngha.med.sa (A.A.); 5 King Saud Bin Abdulaziz University for Health Sciences (KSAU-HS), Riyadh 14811, Saudi Arabia

**Keywords:** antibiotics, overprescription, dental student, attitudes, knowledge, antibiotic resistance

## Abstract

**Background:** The use of antibiotics prophylactically and therapeutically in dentistry has become common practice. Inappropriate prescription may lead to adverse side effects and bacterial resistance. During clinical training, dental students in Saudi Arabia are authorized to prescribe antibiotics. **Aim:** To evaluate dental students’ knowledge and attitudes regarding antibiotic prescription in Riyadh, Saudi Arabia. **Methods:** A cross-sectional study based on a validated questionnaire consisting of 34 questions focusing on antibiotic indications in dentistry, antibiotic regimens, and knowledge regarding resistance was distributed amongst dental students in five leading dental colleges in Riyadh. **Results:** A large proportion of students (71.7%) were familiar with the concept of antibiotic resistance. When comparing junior and senior dental students’ knowledge with regards to indications of antibiotic use in commonly encountered conditions, it was found that there was no significant difference in antibiotic prescription frequency between these groups. Most dental students choose to prescribe amoxicillin as their first-choice of antibiotic (88.4%), and most also chose to use it for a duration of 3–5 days (69.2%). **Conclusions:** This study concludes that dental students may prescribe antibiotics inappropriately to manage various conditions when not indicated. This may indicate a defect in education of students with regards to current antibiotic guidelines.

## 1. Introduction

Since the introduction of penicillin by Alexander Fleming in 1928, antibiotics have become one of the most commonly utilized medication category [1]. The primary function of antibiotics is the eradication of bacterial infections either by killing or slowing down bacterial growth [2]. One of the perhaps unforeseen challenges that faced clinicians after antibiotics had radicalized medicine was the evolutionary measures by which bacteria were able to become resistant to these agents [3].

In dentistry, the use of antibiotics prophylactically and therapeutically has become common practice. Early reports stated that penicillin showed effectiveness in treating cellulitis and angular cheilitis [1]. The most common infections treated with antibiotics in the field of dentistry are infections related to the root canal (endodontic infections). However, the use of antibiotics in such situations is not always warranted. Recent research shows that most endodontic infections could be managed with only local interventions to eliminate or relieve the source of infection, such as incision and drainage, root canal treatment, and tooth extractions, without the need for antibiotic therapy [2,4].

The World Health Organization’s (WHO) Guide to Good Prescribing has recently focused on educating healthcare providers about reasonable prescribing with real-life examples and a step by step guideline [5]. The Faculty of General Dental Practice in the United Kingdom (FGDP UK) and the Scottish Dental Clinical Effectiveness Programme (SDCEP) clinical guidelines have also stated that antibiotics are not recommended in dealing with non-spreading infections of teeth and alveolar bone (dentoalveolar infections) in healthy individuals [6,7]. Many studies have been conducted in the UK, India, and Iran assessing the knowledge of dentists and dental students regarding the prescription and indications of antibiotics, and although the amount of evidence of mounting antimicrobial resistance in medical literature is increasing, studies show that the dental community lacks adequate knowledge in this area [8,9,10,11].

In Saudi Arabia, a 2015 study undertaken in the Northern region concluded that amoxicillin-clavulanate was the most commonly prescribed antibiotic to treat endodontic infections [2]. Furthermore, two nationwide studies were carried out in 2016 and 2017 which found that dentists’ knowledge of antibiotic prescription guidelines and indications was roughly gauged to be intermediate. These studies indicated that there was frequent prescription of antibiotics in situations where they are not necessary or indicated [12,13]. Moreover, a survey that was distributed amongst Riyadh’s non-medical female university students investigating their knowledge and attitudes regarding the medical and dental use of antibiotics revealed that there are misconceptions and a negative attitude towards antibiotics usage in treating dentistry-related problems [14].

As per policy, during the clinical years, dental students in Saudi Arabia are authorized to prescribe antibiotics under the supervision of their instructors [15,16]. Attitudes towards prescribing medications and experience with specific antibiotics usually develop during these early training years and many carry their experiences with them into clinical practice. Thus, it is of fundamental importance that concepts of antibiotic resistance and the indications of prescribing them be instilled early on. The American Association of Endodontists (AAE) has published a guideline on the use of systemic antibiotics and antibiotic prophylaxis. It is the most commonly utilized dental practice guideline for prescribing antibiotics in Saudi Arabia. Its purpose is to provide guidance for the proper use of antibiotic therapy in the treatment of endodontic infections [17,18].

Several studies in the literature have evaluated antibiotic knowledge and prescribing patterns amongst dental practitioners. However, these studies have mostly been concerned with practicing dentists, rather than exploring the knowledge and attitudes of dental students. This study aims to evaluate dental students’ knowledge and attitudes regarding antibiotic prescription guideline and applications in Riyadh, Saudi Arabia.

## 2. Materials and Methods

This is a cross-sectional study that was conducted in Riyadh to assess the knowledge and attitudes of dental students in private and public dentistry schools, with regards to antibiotic prescription in August, 2017.

A validated questionnaire, obtained from a similar study conducted by Konde S et al., was used to assess knowledge and attitudes of dental students [19]. The questionnaire used aided in collecting the following information from study participants: Demographic characteristics, knowledge about antibiotic prophylaxis, knowledge about antibiotic resistance, knowledge of antibiotic prescription guidelines, clinical conditions for which antibiotics are indicated, most commonly prescribed antibiotics, antibiotic regimen durations. The questionnaire contained 34 questions of both multiple choices and closed ended questions. The questionnaire did not require translation since the primary language of instruction in Saudi Arabia is English.

The questionnaire was converted to an electronic form using Google Forms (Google Forms, 2018; a free web-based survey generator). The integrity of the questionnaire was maintained by keeping the options and answering fields as they would appear in paper format. A link to the questionnaire was generated and distributed through social media platforms (Twitter, Telegram, Snapchat, and WhatsApp) to dental students enrolled in the following five dental colleges: King Saud University (KSU), King Saud bin Abdulaziz University for Health Sciences (KSAU-HS), Riyadh Elm University (REU), Princess Nora bint Abdulrahman University (PNU), and Al-Farabi Colleges in Riyadh. Forth year and fifth year students were considered juniors, whereas sixth year students and interns were considered to be seniors. The study’s methodology was reviewed and approved by the Institutional Review Board (IRB) of the King Abdullah International Medical Research Center (KAIMRC). The questionnaire detailed the aim of the study, its design, and the expected utility of its results, thus informed signed consent was not required and participants participated voluntarily.

Collected data was analyzed using SAS^®^ 9.3 Macro Language. For comparison purposes, we compared antibiotic prescription patterns between junior and senior dental students using comparative tests such as the Pearson Chi-square test.

## 3. Results

A total of 312 dental students responded to this survey, 138 of whom were juniors (44.2%), and 174 of whom were seniors (55.7%). Students from Riyadh Elm University represented the largest number of participants at 133 (42.6%) (Table 1). The responses given by the participants regarding antibiotic prescriptions for commonly encountered oral and systemic conditions are presented in (Table 2). When comparing junior and senior dental students, it was found that there was no significant difference in antibiotic prescription frequency between the two groups. However, when questioned with regards to specific conditions, such as localized intraoral swelling, juvenile diabetes, and congenital cardiac abnormalities, there were significant difference between the prescription tendencies of juniors and seniors (*p* = 0.002, *p* = 0.007, and *p* = 0.045, respectively).

A large proportion of senior (72.4%) and junior (71%) dental students were familiar with the concept of antibiotic resistance. As for students’ knowledge of antibiotic prophylaxis, a considerable proportion of juniors (79.7%) and seniors (85.6%) checked that they were aware of the antibiotic prophylaxis guidelines. A significant majority of senior students reported being aware of antibiotic prescription guidelines compared to a lesser proportion of junior students (79.3% versus 66.6%, respectively; *p* = 0.011) (Table 3).

Regarding dental students’ attitude towards prescribing antibiotics, it was found that the vast majority of dental students (88.4%) choose to prescribe amoxicillin as their first-choice of antibiotic. On the other hand, a minority of students, 36 (11.5%), choose to prescribe other antibiotics, including ofloxacin with ornidazole, cephalexin, and clindamycin. The most common duration of an antibiotic course amongst dental students was 3–5 days (69.2%), whereas some students choose to prescribe antibiotics for more than five days (17.6%), and others picked a prescription regimen of three days (13.2%), with no significant difference in durations chosen between juniors and seniors. When assessing the rationale behind an antibiotic prescribing regimen, more than half of the respondents stated that they would prescribe antibiotics based on guidelines (173, 55.4%), while the remainder of students (44.5%) choose to prescribe antibiotics based on the severity of patient’s symptoms (Table 4).

## 4. Discussion

In Saudi Arabia, studies conducted to evaluate the knowledge and attitudes of dental practitioners regarding antibiotic prescription have found that most practitioners lack adequate knowledge in this matter [12,13].

Oral conditions that present to dental clinics are mostly inflammatory conditions which necessitate operative interventions, rather than infectious processes that would benefit from antibiotics [20]. In dentistry, situations that require antibiotic therapy are limited to oral infections accompanied by fever, lymphadenopathy, and trismus [21]. Our study found that dental students in our sample would routinely prescribe antibiotics for conditions that, according to guidelines, do not require them, and which could be managed with operative measures alone [22]. For example, antibiotics were deemed necessary by students for periapical abscess (65.7%), dry socket (37.1%), and pulpitis (25.6%), all of which are conditions which are routinely treated without them (Table 2).

Examples of conditions which have been found to benefit from treatment plans rather than antibiotics include pulpitis and necrotic pulp, for which root canal therapy is considered the standard of care. Antibiotics are frequently prescribed for such conditions although, according to guidelines, their benefit is unproven [17]. A similar situation exists for the commonly encountered situation of dry sockets (alveolar osteitis), which is essentially not an infection and is thus not expected to improve with antibiotics, yet they are frequently prescribed for it [23].

In our study, 40.7% of students stated that they would routinely prescribe antibiotics to handle periodontal diseases. Periodontal disease is not an indication for antibiotic use unless there is a local spreading infection whereby mechanical means, such drainage or debridement, are not possible [24]. Dental extraction is another common procedure in the dental practice where antibiotics administration has showed limited benefit and has been reported to be associated with a higher incidence of gastrointestinal side effects [25]. Participating students in the current study stated that they would mostly prescribe antibiotics for surgical tooth extraction (61.2%), although research has not effectively shown a reduction in postoperative complications (Table 2) [26].

Most of the dental students in our study would consistently prescribe antibiotics for facial swelling (86.2%) as well as draining sinus tracts (62.6%), both of which are not conditions requiring antibiotics. According to the AAE clinical guide, incision and drainage is the primary treatment for pus accumulation within tissues. However, the AAE stated that antibiotics are prescribed in the cases of diffuse swelling (cellulitis), systemic symptoms, or immunocompromised patients [18]. Infection of the operculum, which is known as pericoronitis, is another example of a bacterial infection that is usually treated with local measures. Nearly half of the students in our study thought that pericoronitis is an indication for antibiotic use (49.6%). However, if there is evidence of systemic involvement or persistent swelling despite local measures, a three-day antibiotic course is recommended [27].

In dental practice, penicillin is the most commonly prescribed antibiotic [19]. However, other antibiotics are also reportedly becoming extremely common in the treatment of odontogenic infections, including amoxicillin, penicillin V, metronidazole, and amoxicillin/clavulanate [27]. Our current study showed that amoxicillin is the first choice of antibiotic (88.4%) prescribed by dental students, which is comparable to previous findings reported in India and Iran [9,19,28]. A Study published in Saudi Arabia reported that amoxicillin with clavulanic acid was the most frequently prescribed antibiotic among dental practitioners [12].

The duration of antibiotic course in therapeutic guidelines is commonly based on expert opinions [29]. Prior studies evaluating the average duration of antibiotic course prescribed by dentists in Canada found that dental practitioners prescribe antibiotics for an average of 6.92 days [30]. On the other hand, endodontists in the USA prescribe antibiotics for 7.58 days [31]. According to the British National Formulary (BNF), an antibiotic course of two to three days is advocated for the treatment of acute dento-alveolar infections [20].

Indeed, several reports have shown that patients improved significantly after two to three days of antibiotic therapy, thus proving that prolonged courses may not confer additional benefits [32,33,34,35]. In the present study, only 13.1% of students would prescribe antibiotics for the duration of two to three days, while a great majority of students (86.8%) would place patients on a course of more than three days (Table 4).

Another important finding is that a number of students would prescribe antibiotics for viral infections, noted to be 33.3% of the sample, when in cases of viral infections, such as herpes simplex infection, symptomatic relief is the treatment of choice [36]. As for antibiotic prophylaxis against infective endocarditis (IE), according to the American Heart Association (AHA) and American College of Cardiology (ACC) 2017 guidelines, prophylaxis is suitable for patients undergoing invasive dental procedures, particularly with: Prosthetic cardiac valves, prosthetic material used for cardiac valve repair, previous IE, unrepaired cyanotic congenital heart disease or repaired congenital heart disease, and patients who had cardiac transplant with valve regurgitation due to a structurally abnormal valves [18]. Our study revealed that only 79.1% of students would prescribe antibiotics for patients with known subacute bacterial endocarditis as prophylaxis (Table 2).

Our study has encountered some limitations, which included the fact that amoxicillin with clavulanic acid was not included in the original version of the questionnaire as an option in the most commonly prescribed antibiotics. In addition, since there are no Saudi guidelines for antibiotics, different schools may be teaching different guidelines, thus there may be a baseline difference in these student’s education. Finally, the administration of a cross-sectional questionnaire may have introduced bias whereby student’s practices may reflect those of their mentors, which may or may not be truly evidence-based. Consequently, further studies comparing the attitudes of the mentors and clinical instructors towards antibiotic prescription may shed some light regarding this possible confounding element. 

## 5. Conclusions

Our findings showed that dental students may prescribe antibiotics inappropriately to manage various oral and systemic conditions when they are not indicated. Furthermore, there is a clear defect in education and awareness of students with regards to antibiotic guidelines. Dental diseases are predominantly caused by local factors. The mere removal of the etiological factors reduces the need for prescribing antibiotics, a fact that clearly needs to be reiterated in contemporary dentistry programs. The prescribing practices of dental students can be improved by emphasizing the recommended guidelines in dental schools’ curricula and clinical manuals. Further studies are needed to truly elucidate the effect of a guideline-focused education on students’ prescription patterns.

## Figures and Tables

**Table 1:** Demographic profile of the participants.

Demographic Characteristics of the Study Cohort	*n* (%)
Gender:	
*Female*	169 (54.17)
*Male*	143 (45.83)
Colleges:	
*Riyadh Elm University*	133 (42.63)
*Al-Farabi Colleges*	67 (21.47)
*King Saud bin Abdulaziz University for Health sciences*	47 (15.06)
*King Saud University*	33 (10.58)
*Princess Nora bint Abdulrahman University*	32 (10.26)
School year:	
*Fourth-year*	41 (13.14)
*Fifth-year*	97 (31.09)
*Sixth-year*	119 (38.14)
*Intern*	55 (17.63)

**Table 2:** Comparison of the number of affirmative responses that the two groups of students, juniors and seniors, gave for different clinical scenarios.

Conditions	Juniors *n* (%)	Seniors *n* (%)	*p*-Value
Reversible pulpitis	26 (18.84)	46 (26.44)	0.113
Irreversible pulpitis	32 (23.19)	56 (32.18)	0.079
Intraoral draining sinus tract	78 (56.52)	110 (63.22)	0.229
Extraoral draining sinus tract	91 (65.94)	112 (64.37)	0.772
Localized intraoral swelling	59 (42.75)	105 (60.34)	0.002 *
Acute facial swelling	124 (89.86)	145 (83.33)	0.097
Dental trauma	36 (26.09)	62 (35.63)	0.071
Periodontal diseases	50 (36.23)	77 (44.25)	0.152
Pericoronitis	72 (52.17)	83 (48.26)	0.492
Simple extraction	11 (7.97)	26 (14.94)	0.058
Extraction by open method	88 (63.77)	103 (59.20)	0.410
Periapical abscess	86 (62.32)	119 (68.39)	0.261
Apical periodontitis	49 (35.51)	60 (34.48)	0.850
Dry socket	53 (38.41)	63 (36.21)	0.689
Viral infections	44 (31.88)	60 (34.48)	0.628
Juvenile diabetes	45 (32.61)	83 (47.70)	0.007 *
Blood dyscrasias	52 (37.68)	77 (44.25)	0.241
Respiratory disorders	46 (33.33)	60 (34.48)	0.831
Congenital cardiac abnormalities	58 (42.03)	93 (53.45)	0.045 *
Subacute bacterial endocarditis	107 (77.54)	140 (80.46)	0.527

* Statistical significant difference between the groups; *p* ≤ 0.05.

**Table 3:** Knowledge of antibiotic resistance and prescription guidelines.

Questions	Juniors *n* (%)	Seniors *n* (%)	*p*-Value
Knowledge of antibiotic resistance	98 (71.01)	126 (72.41)	0.785
Knowledge of antibiotic prophylaxis	110 (79.71)	149 (85.63)	0.166
Knowledge of the guidelines for antibiotic prescription	92 (66.67)	138 (79.31)	0.011 *

* Statistical significant difference between the groups; *p* ≤ 0.05.

**Table 4:** Dental student’s antibiotic prescription patterns and regimens.

Antibiotic Prescription Patterns	Juniors *n* (%)	Seniors *n* (%)	*p*-Value
Most common antibiotic prescribed:			
*Amoxicillin*	121 (87.68)	155 (89.08)	0.571
*Clindamycin*	5 (3.62)	3 (1.72)	
*Other*	12 (8.70)	16 (9.20)	
Duration of antibiotic course:			
*<3 days*	17 (12.32)	24 (13.79)	0.830
*3–5 days*	98 (71.01)	118 (67.82)	
*>5 days*	23 (16.67)	32 (18.39)	
Decision of prescribing Antibiotics:			
*Based on symptoms*	60 (43.48)	69 (39.66)	0.781
*Based on guidelines*	74 (53.62)	99 (56.90)	
*Based on the cost of the drug*	4 (2.90)	6 (3.45)	
